# Building medical oxygen systems in a resource-limited setting: the case of Cameroon

**DOI:** 10.7189/jogh.15.04087

**Published:** 2025-02-14

**Authors:** Yauba Saidu, Clarence Mbanga, Sandra Mokom, Andreas Frambo, Ousmane Diaby, Audrey Battu, Zakary Katz

**Affiliations:** 1 Clinton Health Access Initiative, HS Jean-Paul II Boulevard, Tsinga sous-prefecture, Yaoundé, Cameroon; 2 Institute for Global Health, Santa Chiara Lab, University of Siena, Siena, Italy; 3 College of Public Health and Health Professions, University of Florida, Florida, USA; 4 Faculty of Economics and Management, University of Ngaoundere, Cameroon; 5 Global Essential Medicines, Clinton Health Access Initiative Inc., Boston, Massachusetts, USA

## Abstract

**Background:**

Medical oxygen is of critical importance in resource-limited settings where hypoxaemia-causing conditions are prevalent. Its availability/accessibility is, however, often hindered by numerous challenges. Here we present concerted efforts that we undertook to optimise the medical oxygen system in Cameroon in a bid to enhance patient care and reduce hypoxaemia-related mortality.

**Methods:**

Technical and financial support was provided to the Cameroonian Ministry of Public Health across three key areas. First, we set up a governance framework via the establishment of two multi-sectoral technical working groups to develop strategies and governing standards for medical oxygen production and use in Cameroon. We then quantified the country’s medical oxygen needs by leveraging the results of a two-phase health facility assessment. Lastly, we implemented additional systems ensuring effective and real-time monitoring of medical oxygen investments, and we coordinated the working groups to mobilise funding from different sources.

**Results:**

Efforts in developing a governance framework for medical oxygen resulted in the development of a National Strategic Plan for the Provision of Medical Oxygen in Cameroon (2021–2025) centred around four key areas: policy and financing, availability and maintenance of medical oxygen supply systems, clinical management of hypoxaemia, and data collection and use for hypoxaemia management. The second output was a norms and standards document on the production and use of medical oxygen in Cameroon. Quantification revealed a considerable gap in medical oxygen equipment and a monthly medical oxygen demand of about 162.4 million litres, the majority required by district hospitals. To begin bridging the identified gap in equipment, we procured and distributed medical oxygen equipment to 67 health facilities across the country. Furthermore, we successfully supported the government in securing funding from the Global Fund to install five large pressure swing absorption plants in five strategically located hospitals, as well as setting up a liquid oxygen tank and piping system in one high-volume regional hospital. Furthermore, we set up a system to enable effective monitoring and use of medical oxygen facilities, which comprised the definition of 15 indicators and their subsequent integration into the District Health Information System 2 (DHIS-2). Lastly, we trained 601 healthcare personnel on hypoxaemia diagnosis, treatment, and DHIS-2 reporting.

**Conclusions:**

Our concerted efforts with the Ministry of Public Health have yielded significant benefits in the establishment of a sustainable medical oxygen system in Cameroon, further strengthening the country’s emergency response mechanism. However, considerable gaps remain, highlighting the need for sustained collaboration between the government, private partners, and international organisations for resource mobilisation.

Hypoxaemia, a physiological condition characterised by inadequate blood oxygen levels, often arises as a consequential complication of diseases that impede ventilation and gas exchange, such as chronic obstructive pulmonary disease (COPD), asthma, pulmonary embolism, and post-operative states [1]. It can also result from diseases characterised by increased oxygen demand such as COVID-19, meningitis, perinatal asphyxia, severe sepsis, and severe anaemia resulting from road traffic accidents or malaria amongst others [2]. Regardless of the cause and other clinical or laboratory factors, hypoxaemia significantly increases the likelihood of poor health outcomes, particularly death [3], underlining the need for prompt diagnosis and effective management.

Medical oxygen therapy plays a vital role in the management of hypoxaemia and has been shown to improve patient outcomes and reduce mortality rates associated with the condition [4]. The benefits of medical oxygen therapy extend to various clinical scenarios, including the improvement of clinical and biological parameters of patients with persistent acute respiratory failure [5]; the prevention of postoperative hypoxaemia resulting from incomplete lung re-expansion, reduced chest wall and diaphragmatic activity, residual effects of anaesthetic drugs [6]; and the reduction of childhood pneumonia mortality in high-burden settings [7,8]. Despite these proven benefits of medical oxygen therapy and evidence suggesting the cost-effectiveness of investments in medical oxygen systems [9], access to this life-saving commodity is still constrained by infrastructural and financial challenges, particularly in resource-limited settings.

One such setting is Cameroon, where hypoxaemia-causing conditions remain prevalent, contributing significantly to high mortality, underscoring the critical importance of ensuring uninterrupted access to medical oxygen. This challenge was further exacerbated by the COVID-19 pandemic, which increased the risk of patients developing hypoxaemia and experiencing severe respiratory complications that require medical oxygen therapy [10]. However, availability and accessibility to medical oxygen in the country are hindered by several challenges, such as limited infrastructure, inadequate maintenance of existing facilities, insufficient personnel training, unreliable electricity supply and financial constraints [11]. Addressing these challenges requires a comprehensive approach that will include the optimisation of oxygen sources, distribution networks, storage equipment, and delivery devices tailored to the specific needs of the context. Healthcare personnel training, regular maintenance, and quality control measures are also needed to ensure optimal patient care and long-term sustainability of oxygen investments [12]. Establishing such systems is not only cost-effective, but can also save lives, reduce morbidity, and strengthen the overall healthcare system, thus preparing it to appropriately respond to public health emergencies requiring medical oxygen.

Between 2021 and 2023, we spearheaded efforts to lay the groundwork needed to establish a sustainable medical oxygen system in Cameroon. Specifically, we collaborated with key departments in the Ministry of Public Health (MoPH) to develop a governance framework for medical oxygen production and use in Cameroon, quantify the medical oxygen needs of the country to guide future investments, mobilise financial resources, and set up additional systems to monitor the impact of medical oxygen investments and improve medical oxygen availability and accessibility. Here we present our approach to and the results from these concerted efforts to provide insights that could inform the set-up of medical oxygen systems in other similar resource-limited settings, especially in sub-Saharan Africa.

## METHODS

### Study setting

We conducted this study in Cameroon, a country located in the west-central part of Africa. It is administratively divided into 10 regions and has a population of about 30 million inhabitants [13]. Its healthcare system, meanwhile, is organised into central, intermediate, and peripheral levels, and consists of over 6000 health facilities spread across 203 health districts [14]. These facilities are classified across six distinct categories (general hospitals, central hospitals, regional hospitals, district hospitals, medicalised health centres, and integrated health centres). The system currently faces several infrastructural and economic challenges that significantly impact its health service delivery. In terms of infrastructure, aside from being insufficient in numbers, many health facilities are also poorly equipped, with limited personnel and access to essential medical supplies and technologies. The country struggles with inadequate transportation networks, which hinder the distribution of oxygen cylinders and other critical resources, especially in rural and remote areas. Economically, Cameroon experiences constraints such as high poverty levels and limited public investment in its health system. These factors contribute to a shortage of trained healthcare professionals and affect service delivery and the overall quality of care [15].

### Study procedure

#### Development of a governance structure for medical oxygen production and use in Cameroon

Our first step was to engage the leadership at the MoPH to set up a multisectoral technical working group consisting of experts from various stakeholder departments (directorates of the MoPH, university teaching hospitals, development partners, civil society, *etc*.), tasked with the responsibility of designing and implementing a rapid, nation-wide situational analysis to quantify and characterise gaps in the medical oxygen ecosystem in Cameroon. The situational analysis was conducted between May 26 to 1 June 2021 in 114 health facilities (62 district hospitals, 12 regional hospitals, 10 central hospitals, 5 general hospitals, and 25 private/confessional health facilities assimilated to the level of district hospitals) across all ten regions of the country. The objectives of the assessment were to assess the availability and use of medical oxygen policy documents and guidelines; evaluate the financing of medical oxygen; assess the availability of medical oxygen; determine the availability and functionality of oxygen equipment and devices; and evaluate the data management system for the management of hypoxaemia/hypoxia. Findings from the situational analysis were leveraged by the technical working group to identify major bottlenecks in the provision and use of medical oxygen in Cameroon and subsequently develop tailored strategies that could mitigate these challenges and dramatically improve hypoxaemia management and medical oxygen availability and accessibility in the short, medium, and long terms. These strategies were packaged into a strategic document that would be used by the MoPH as an advocacy and resource mobilisation tool, as well as a decision-making guide on its medical oxygen investments.

We further set up a technical team made up of key personnel from different directorates of the MoPH (Department of Health Care Organization and Technology, Directorate of Pharmacy, Medicines and Laboratories, Cooperation Division, Legal Affairs and Litigation Division, Studies and Projects Division), experts from general and central hospitals (Yaoundé Emergency Centre, Yaoundé Central Hospital, Yaoundé Gynaecology, Obstetrics and Paediatrics Hospital), the General Delegation for National Security, development partners (World Health Organization (WHO), United Nations Children’s Fund (UNICEF), the United States Centers for Disease Control Cameroon country office, *etc*.), the Catholic Health Organization in Cameroon, and civil societies, to draft governing standards for medical oxygen production, storage, distribution and use in the country. The essence of setting up this multisectoral team was to ensure that the norms and standards document captured the opinions of all key players in the medical oxygen space. We then organised a series of working sessions for the technical team to finalise and validate these standards.

#### Quantification of medical oxygen needs

We further supported the MoPH in quantifying the country’s medical oxygen needs to inform future investments geared towards improving the medical oxygen production capacity. The first step in this process involved conducting an assessment to obtain baseline information on the characteristics and oxygen consumption needs of different wards/departments across the different health facility types in the country. This was done in two phases using the revised and adapted version of the WHO Biomedical Equipment Inventory tool, with the first phase carried out between November 22 and 6 December 2022, in 99 health facilities and functional COVID-19 treatment centres across the 10 regions, and the second phase conducted from 16 May to 15 July 2022 in 60 of the 99 centres used during the first phase, to collect additional data that was omitted during the first phase.

With baseline data on health facility characteristics obtained via the assessment, the next step in the quantification process involved estimating the proportion of beds, bed turnover, and average occupancy rate per ward/department per facility type. Given the unavailability of patient admission information from the health facilities sampled during the assessment, we derived these estimates using existing literature and evidence from other sub-Saharan African countries with comparable facility types [16–18] (**Table 1**). These assumptions were considered valid for Cameroon because of comparable demographic and health profiles, as well as shared infrastructural challenges between Cameroon and other sub-Saharan countries from which the assumptions were pulled. Using the estimated number of beds per facility, bed turnover and average occupancy rates, alongside clinical assumptions (hypoxaemia prevalence, medical oxygen flow rate and therapy duration for a typical case of hypoxaemia per ward/department) from the UNICEF Oxygen System Planning Tool [19] (**Table 2**), we then computed the annual medical oxygen need per ward/department per facility type. This was obtained by multiplying the medical oxygen needed for one patient by the number of hypoxemic patients per year, per ward/department (**Table 3**).

**Table 1:** Annual bed turnover rate*

	Number	Number of beds	Annual bed turnover rate
**General and similar hospital**	4	919	41
**Central and similar hospitals**	13	2595	96
**Regional hospital**	15	2487	72
**District hospital**	67	5090	58
**Total**	99	11 111	Mean = 66.75

*Source: health management information system, July 2019 to June 2020

**Table 2:** Clinical assumptions for estimating oxygen needs*

Ward/department	Prevalence of hypoxaemia by bed type (%)	Typical oxygen flow rate (litres per minute)	Duration of oxygen therapy (in hours) for a typical case of hypoxaemia
**Outpatient department**	0	5	1
**Adult/internal medicine**	6	6	144
**Paediatrics**	10	2	72
**Neonatology**	20	2	72
**Intensive care**	100	6	96
**Operating theatre**	100	8	6
**Emergency**	40	6	16
**Maternity**	13	5	12
**Recovery**	100	5	6

*Source: UNICEF Oxygen System Planning Tool [19].

**Table 3:** Example of oxygen need quantification

	Oxygen needs for one patient (in litres)	Number of hypoxemic patients per year	Annual oxygen need (in litres)
	**A**	**B**	**A × B**
**Neonatal ward**	1 L/min × 3 days of oxygen therapy = 4320 L	20% hypoxaemia prevalence × 10 beds × 50 (annual bed turnover rate) = 100 hypoxaemic patients	432 000
**COVID ward (adult severe patient)**	10 L/minute × 7 days of oxygen therapy = 100 8000 L*	100% hypoxaemia prevalence × 5 beds × 25 (annual bed turnover rate) = 125 hypoxaemic patients†	12 600 000

*Source: national stakeholder input, WHO guidelines, and literature review.

†Source: DHIS-2 data and literature review.

We summed up the annual medical oxygen needs of different wards/departments for each facility type and multiplied it by the total number of facilities of the same type to obtain the total annual medical oxygen need of that facility type. This was done for all the different facility types (**Table 1**). Finally, we summed up the total requirements across all the different facility types to obtain the overall medical oxygen needs for the entire country.

#### Additional systems to improve medical oxygen availability and accessibility

Given the considerable financial investment that building sustainable medical oxygen systems represents, reliable systems had to be established to ensure that the country has the necessary financial resources, that healthcare workers can diagnose and treat hypoxaemia effectively, and that the impact of medical oxygen-related investments is monitored in real-time to guide decision making, resource allocation, and future investments. To this end, we assisted the MoPH in defining key indicators for hypoxaemia management and medical oxygen availability/accessibility, as well as the integration thereof the national health information management system, the District Health Information System – 2 (DHIS-2). We also supported the MoPH in training healthcare workers across the 10 regions of the country on the diagnosis and treatment of hypoxaemia/hypoxaemia, as well as on the capture and reporting of resulting data. Finally, we organised a series of workshops to develop funding proposals, targeting COVID-19 Country Response Mechanism funds from the Global Fund.

## RESULTS

### Development of governance structure for medical oxygen production and use in Cameroon

The rapid situational analysis identified a lack of policies and guidelines on the use of medical oxygen, insufficient availability of equipment, materials, and consumables for the diagnosis and management of hypoxaemia, inadequate systems for collecting and transmitting data on the use of medical oxygen, and insufficient funding for medical oxygen, as principal bottlenecks to medical oxygen availability and accessibility in Cameroon. These findings, which are available elsewhere [20], were leveraged by the technical working group to develop a National Strategic Plan for the Provision of Medical Oxygen in Cameroon (2021–2025), which focusses on four key areas of intervention to drastically improve the availability and access to medical oxygen for the management of hypoxaemia over the short, medium, and long term: policy and financing, the availability and maintenance of diagnostics and medical oxygen supply systems, the clinical management of hypoxaemia, and data collection and use for hypoxaemia management. The plan was endorsed by the Cameroon Minister of Public Health during a high-level meeting organised in Yaoundé, Cameroon. The total cost of the plan was estimated at USD 219 775 735.

A comprehensive document on the norms and standards for the production, storage, distribution, and use of medical oxygen in Cameroon was also developed, detailing standards for medical oxygen production, storage and distribution; medical device standards, norms, and standards for medical oxygen use; quality management for the production, storage, distribution, and use of medical oxygen; and monitoring and evaluation standards for medical oxygen investments to ensure the quality and safety of medical oxygen therapy in hospital and pre-hospital settings. It was publicly adopted and validated during a high-level meeting presided over by the Director of Health Care Organization and Technology at the MoPH in June 2022, in Yaoundé, Cameroon.

### Quantification of medical oxygen needs and strategies to meet identified needs

#### Quantification of medical oxygen needs

**Figure 1:**
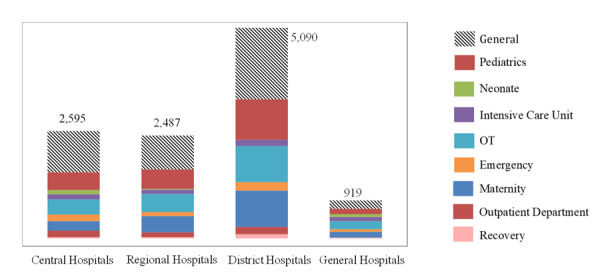
Number of beds per ward/department per facility type.

From the pre-quantification assessment, we estimated that the country’s health facilities had 11 111 beds, with almost half (45%) being in district hospitals. The beds were mostly found within the general, paediatrics, maternity and operating theatre wards (**Figure 1**).

**Figure 2:**
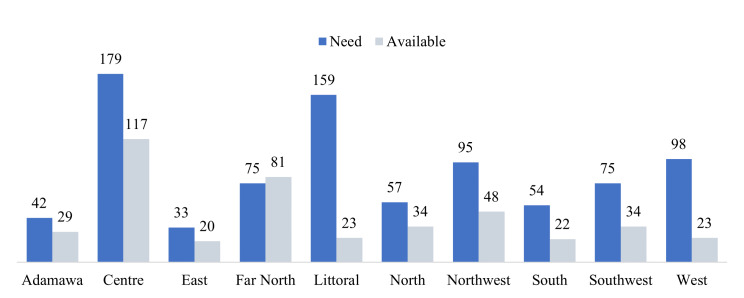
Oxygen concentrators availability vs required per region.

Regarding the oxygen production sources such as concentrators, most (93%) facilities had at least one functional oxygen concentrator. However, their distribution was inequitable, with regions like the Far North having 8% more concentrators than needed, compared to the Littoral region which only had 14% of its needs in oxygen concentrators met. Overall, only six of the country’s ten regions had over 50% availability of oxygen concentrators (**Figure 2**).

**Figure 3:**
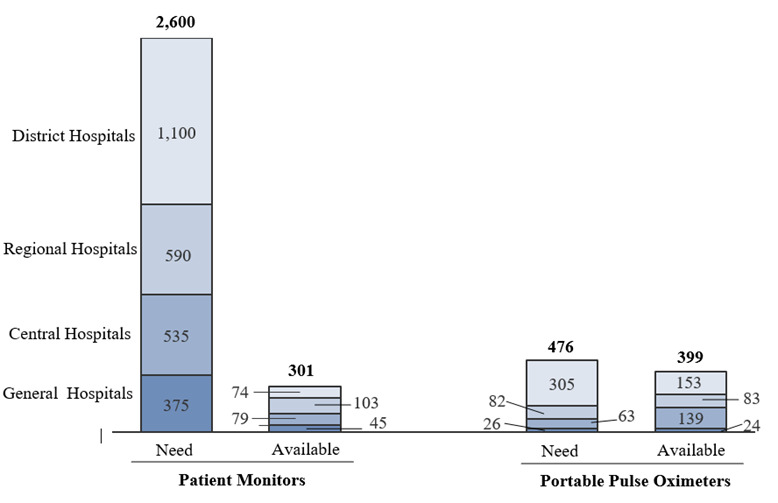
Patient monitors and pulse oximeter availability vs required per facility type.

Patient monitors and pulse oximeters – crucial devices for diagnosing hypoxaemia and consequently determining the need for medical oxygen therapy – were not sufficiently available. While 80% of facilities were found to have at least one functional monitor, the total number of functional monitors represented only about 12% of estimated needs. The availability of pulse oximeters varied considerably across facility types, with only 50% of needs met for district hospitals, as opposed to general and central hospitals, whose supply exceeded and doubled their needs, respectively (**Figure 3**).

**Figure 4:**
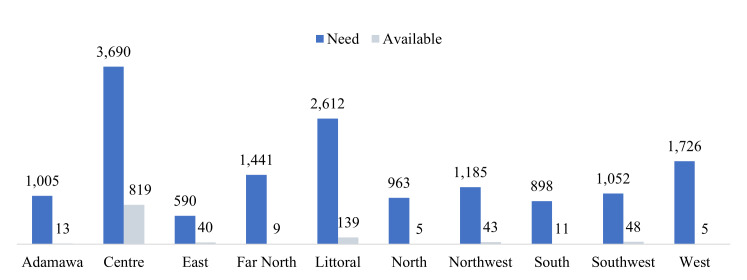
Oxygen cylinder availability (in 10 000 L) vs need per region.

**Figure 5:**
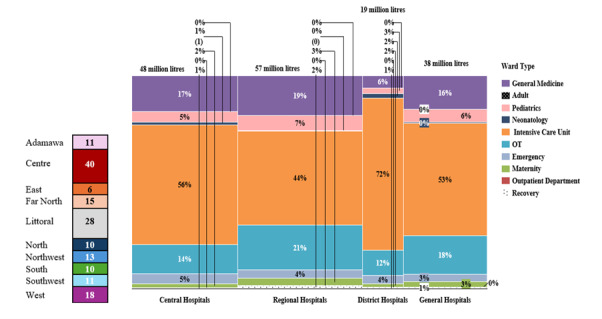
Distribution of medical oxygen demand by region, facility type, and department.

There was a significant shortage of oxygen cylinders in all regions, with 54 cylinder-needing facilities found to have no oxygen cylinders available. This shortage was most pronounced in the West region, where available cylinders met less than one per cent (0.3%) of the total demand. Overall, the shortage in cylinders represented a gap of about 140 million litres, equivalent to 18 700 large (7500 L) cylinders (**Figure 4**). The total monthly medical oxygen demand was estimated at 162.4 million litres, with the greater share required by district hospitals (**Figure 5**).

#### Strategies to meet identified needs

To begin bridging the considerable gap in medical oxygen equipment underlined by the quantification exercise, we procured and distributed medical oxygen equipment and consumables, including 1300 cylinders, 70 oxygen concentrators, 160 pulse oximeters, 250 regulators, 700 humidifiers, 500 flowmeters, 2200 oxygen masks, 15 000 nasal cannulas, 6000 venturi masks, and 860 self-inflating resuscitation bags with masks to 67 health facilities across the country, thanks to catalytic funding from Unitaid. These facilities were selected based on their needs, as determined through an iterative consultative process with the MoPH.

To boost the availability of medical oxygen in reference facilities, we led a series of works at the Limbe Regional Hospital (a referral, tertiary-level hospital in the Southwest region of the country) geared toward the installation and commissioning of a state-of-the-art vacuum-insulated evaporator tank and vaporiser for storing and converting the liquid oxygen into a gaseous state for safe and efficient supply to patients in need. This necessitated the procurement of the tank and its accessories, the performance of civil works (electrical connections, structural enhancements) aimed at adapting the existing infrastructure to accommodate the vacuum-insulated evaporator system, and the installation of a dedicated network of pipelines and valves to enable the safe and efficient distribution of medical oxygen throughout the hospital to ensure its availability at the point of care.

The choice of Limbe Regional Hospital for this investment was guided by two primary reasons. First, being a large reference hospital in the region, it appeared to have a high oxygen need. Second, it is located less a 100 km away from a major private medical oxygen production facility that could easily deliver liquid oxygen to the site. A tripartite medical oxygen delivery contract has been established among Clinton Health Access Initiative, the hospital and the private medical oxygen producer and the contract is expected to run for 24 months, from January 2025 to 31 December 2026.

This investment in Limbe complements other investments previewed in the strategic plan, including the installation of five large pressure swing absorption (PSA) plants in five of the country’s 10 regions. These five regions are located hundreds to thousands of kilometres from the private oxygen production plan in the economic capital of Douala, making the delivery of liquid oxygen a more costly intervention. Funding for the procurement of these PSA plants (USD 25 million) was secured from the Global Fund’s COVID-19 Country Response Mechanism thanks to a series of workshops we organised, alongside technical meetings with the Global Fund. The PSAs have already been procured and delivered in Cameroon and civil works have commenced in the target facilities. Our future interest would be to compare the cost-effectiveness of each model and thus provide solid evidence to guide decision-making in oxygen investments.

Furthermore, we led the development of a comprehensive plan for the rehabilitation of a defective PSA plant (to be implemented by Build Health International) to help boost the country’s medical oxygen production capacity and move it a step closer towards meeting medical oxygen needs over the long term.

### Additional systems to improve medical oxygen availability and accessibility

To promote evidence-based decision-making and planning around medical oxygen-related needs, optimise resource allocation, and enhance the overall response to medical oxygen needs, ultimately contributing to better patient care and health outcomes, we developed, tested, and integrated key indicators for tracking the use of medical oxygen in the management of hypoxia/hypoxaemia into DHIS-2 (**Table 4**).

**Table 4:** Hypoxaemia management and medical oxygen use indicators integrated into DHIS-2

Indicator
Percentage of HFs with a hypoxaemia/ hypoxia management department
Average number of stock-outs days of medical oxygen during the quarter
Total quantity of medical oxygen available at the beginning of the month
Total quantity of medical oxygen received during the month
Total quantity of medical oxygen consumed during the month
Total quantity of medical oxygen lost or damaged during the month
Proportion of functioning pulse oximeters during the month
Proportion of functioning pulse oximeters during the month
Proportion of 1st, 2nd, 3rd, and 4th category HFs with functioning respirators
Proportion of 1st, 2nd, 3rd, and 4th category HFs with a functioning (pressure swing absorption/air separation unit) oxygen generator
Total 7500 L medical oxygen cylinders (containing medical oxygen or not)
Proportion of health workers who received capacity building in hypoxaemia management
Proportion of patients affected by hypoxaemia/hypoxia who received oxygen therapy
Therapeutic success rate

HF – health facility

We further provided support in the training of 291 district and facility data managers on the collection and entry of medical oxygen-related data into DHIS-2 through a three-day capacity-building workshop covering the basics of DHIS-2, reporting and analysis of medical oxygen-related data, and effective data navigation for decision-making. As of quarter three of 2023, 1377 health facilities were already reporting on medical oxygen use and hypoxia/hypoxaemia management. We equally supported the training of 310 healthcare staff from districts across all ten regions on the diagnosis and management of hypoxia/hypoxaemia.

We also provided a series of trainings on how to ensure the safe, effective operation and longevity of new investments in PSA plants. Specifically, we first rained 29 MoPH leaders and selected senior hospital managers via a two-day capacity-building session on key aspects, including staffing, the supply chain of consumables, spare and replacement parts, skilled external support, and appropriate infrastructure. Furthermore, we trained 35 biomedical engineers, technicians, facilities managers, and plant operators through a five-day workshop on PSA plant maintenance and management, providing them with the knowledge, skills, and materials necessary to maintain the uninterrupted operation of the PSA plants at their facilities. These capacity-building initiatives, which also included a site visit to a PSA plant in Yaoundé for practical, hands-on learning, enhanced the project's sustainability, minimising the need to install new PSA plants whenever one malfunctioned.

## DISCUSSION

Here we describe our collaborative efforts with the Cameroon MoPH to lay the groundwork for establishing a sustainable medical oxygen system in Cameroon, as well as the resulting achievements. Specifically, we supported the MoPH in developing a governance framework for medical oxygen production and use in Cameroon, quantifying the medical oxygen needs of the country to guide future investments, and setting up additional systems to monitor the impact of medical oxygen investments and improve medical oxygen availability and accessibility. Key achievements from these concerted efforts include the development of a National Strategic Plan for the Provision of Medical Oxygen in Cameroon (2021–2025); the development of a norms and standards document for medical oxygen production and use in Cameroon; the quantification of the country’s medical oxygen needs and initial efforts to meet identified gaps; the definition and integration of key indicators of medical oxygen use and hypoxaemia management into DHIS-2; and the training of over half a thousand healthcare personnel on the diagnosis and treatment of hypoxaemia, as well as the reporting of resulting data into DHIS-2.

Through the development of the National Strategic Plan for the Provision of Medical Oxygen in Cameroon (2021–2025) and the norms and standards document, the country was able to clearly define its vision and strategic direction regarding medical oxygen, which not only guides government investments in the medical oxygen space, but also provides a clear roadmap for technical and financial partners to support the country in its mission of making medical oxygen available to all those in need of it. The norms and standards document on the production, storage, distribution and use of medical oxygen represented a significant step in the regulation of medical oxygen production and use in Cameroon. Setting up such a regulatory framework is crucial to ensuring that medical oxygen meets rigorous quality standards, as well as guaranteeing its purity and safety for patient use. It also enables effective monitoring and control of the supply chain, minimising shortages and disruptions in supply and ensuring patients' continued access to this life-sustaining resource. It equally promotes accountability by providing clear guidelines and standards for manufacturers, distributors, and healthcare providers involved in the production, storage, distribution, and use of medical oxygen. This ensures adherence to responsible practices and established protocols, while reducing the risks associated with fraudulent or substandard products and inappropriate use.

We further supported the MoPH in quantifying Cameroon’s medical oxygen needs, which was of high importance for several reasons. First, it allowed for the identification of gaps related to medical oxygen production capacity and medical oxygen equipment and consumables. This further enabled the effective healthcare planning required to ensure that medical oxygen is available at critical points of care and that health facilities are adequately equipped to meet the oxygen demands of patients – two critical factors required to improve patient outcomes and save lives. Second, the quantification served as a valuable tool for guiding optimal resource allocation and infrastructure investment decisions, as it provided insights into areas that may require additional oxygen production plants, storage facilities, or distribution networks. This was leveraged in the targeted procurement and distribution of medical oxygen equipment and consumables to specific health facilities across the country and in the development of alternative solutions to meet identified gaps, including the development of a rehabilitation plan for a defective PSA plant and the need to set-up an oxygen production plant at the Limbe Regional Hospital, as well as the installation of large PSA plants in strategically located health reference facilities. Such strategic investments would definitely help to establish a robust medical oxygen ecosystem that not only enhances the resilience and responsiveness of the Cameroonian healthcare system to disease outbreaks like COVID-19 or other natural disasters, but ultimately contributes to overall health system strengthening that guarantees uninterrupted supply of medical oxygen to patients suffering from hypoxaemia from common, but life-threatening conditions like malaria, sepsis, shock and haemorrhage from post-partum and road traffic accidents.

Our efforts in setting up additional systems to improve medical oxygen availability and accessibility resulted in the integration of key indicators of medical oxygen use and hypoxaemia management into DHIS-2. This achievement brings significant benefits to Cameroon's healthcare system, as it enables systematic monitoring and evaluation of medical oxygen interventions and investments. This information could be leveraged by healthcare administrators and policymakers for needs assessments and optimal resource allocation to ensure a reliable and uninterrupted supply of medical oxygen and related equipment and consumables. Moreover, the integration of these indicators into the DHIS-2 may enhance research and policy development, which in turn may contribute to evidence-based interventions, practices, and guidelines to improve hypoxaemia diagnosis and management, as well as medical oxygen use in Cameroon. Aside from integration, building the capacity of healthcare workers on hypoxaemia management and the reporting of resulting data into DHIS-2 may improve their skills and the overall quality of care delivered in the process, ultimately resulting in more positive treatment outcomes for patients requiring medical oxygen. This assumption is supported by findings from a before-and-after interventional study in Nigeria, which showed an increase in the proportion of pneumonia patients receiving a pulse oximetry and oxygen therapy two months following staff training after the deployment of oxygen equipment [21]. Collectively, improving access to medical training for healthcare workers on the use of diagnostic equipment such as pulse oximetry and providing them with the necessary guidelines has the potential to prevent needless deaths in many low-resource settings

### Limitations

Though the estimates derived using existing literature and evidence from other sub-Saharan African countries with similar healthcare facility types are considered valid for Cameroon, we acknowledge that this approach has limitations, including potential differences in disease prevalence and healthcare practices, which may affect the generalisability of our findings.

## CONCLUSIONS

Building medical oxygen systems in resource-limited settings is a pressing and critical task that necessitates long-term commitment, strategic planning, and a multifaceted approach to bridge existing gaps and ensure the availability of quality medical oxygen for all patients in need. Investing in medical oxygen systems is key to preparing the health system for future pandemics and has the potential to significantly improve healthcare outcomes and save numerous lives. The concerted efforts described here have laid the groundwork necessary to build a sustainable medical oxygen ecosystem in Cameroon. Despite this, current investments remain insufficient to bridge existing gaps in medical oxygen production to meet demand. This emphasises the importance of collaborative efforts between the government, private partners, and international development organisations to mobilise the necessary resources and expertise needed to scale up investments and bridge the gap between medical oxygen production and demand. In this light, we will continue to explore mechanisms to support Cameroon’s MoPH in scaling current medical oxygen investments and mobilising resources required for the effective operationalisation of its strategic plan for the provision of medical oxygen.
